# Structural Investigation of Cell Wall Xylan Polysaccharides from the Leaves of Algerian *Argania spinosa*

**DOI:** 10.3390/molecules21111587

**Published:** 2016-11-21

**Authors:** Kadda Hachem, Céline Faugeron, Meriem Kaid-Harche, Vincent Gloaguen

**Affiliations:** 1 Laboratoire de Biotoxicologie, Pharmacognosie et Valorisation Biologique des Plantes (LBPVBP), Département de biologie, Faculté des sciences, Université Dr. Tahar Moulay de Saida, BP 138 cité ENNASR, 20000 Saida, Algérie; 2 Laboratoire des Productions, Valorisations Végétales et Microbiennes (LP2VM), Département de Biotechnologie, Faculté des sciences de la nature et de la vie, Université des Sciences et de la Technologie d’Oran, BP 1505 El M’Naouar, 31000 Oran, Algérie; kaidharche@yahoo.fr; 3 Laboratoire de Chimie des Substances Naturelles (EA1069), Faculté des Sciences et Techniques, Université de Limoges, 123 avenue Albert Thomas, 87060 Limoges, France; celine.faugeron@unilim.fr (C.F.); vincent.gloaguen@unilim.fr (V.G.)

**Keywords:** *Argania spinosa*, leaf, xylan, MALDI-TOF MS

## Abstract

Xylan-type polysaccharides were isolated from the leaves of *Argania spinosa* (L.) Skeels collected in the Tindouf area (southwestern Algeria). Xylan fractions were obtained by sequential alkaline extractions and purified on Sepharose CL-4B. The xylan structure was investigated by enzymatic hydrolysis with an endo-β(1→4)-xylanase followed by chromatography of the resulting fragments on Biogel P2, characterization by sugar analysis and matrix-assisted laser desorption ionization-time of flight mass spectrometry (MALDI-TOF MS ). The results show that the *A. spinosa* xylan is composed of a β-(1→4)-d-xylopyranose backbone substituted with 4-*O*-methyl-d-glucuronic acid and L-arabinose residues.

## 1. Introduction

In Algeria, many plant species grow spontaneously, despite weather conditions that are not always favorable. These plants colonize a fairly large area of the Sahara region and provide a source of molecules, particularly polysaccharides including hemicellulose, that the food, cosmetic, and pharmaceutical industries exploit for their remarkable properties.

Hemicelluloses constitute, after cellulose, the second most abundant polysaccharide in the plant kingdom. Hemicelluloses are alkaline-soluble polysaccharides with varied content comprising pentoses (d-xylose and l-arabinose), hexoses (d-glucose, d-galactose, l-fucose, d-mannose, and l-rhamnose), and uronic acids (d-glucuronic and 4-*O*-methyl-d-glucuronic acids) [1]. Hemicelluloses of plant cell walls include xyloglucans, xylans, mannans (galacto-/gluco-mannans), glucuronomannans, and mixed glucans [2]. Xylans are the most abundant hemicellulose-type polysaccharides found in plants. The structural diversity of xylans depends on the phylogenetic position of the species, the varietal origin, the tissue or cell type, and the developmental age of the cells [3].

In this context, we were interested in cell wall hemicelluloses from the leaves of the Algerian argan tree. This remnant of former tropical vegetation, belonging to the Sapotaceae family, is well adapted to extreme conditions of aridity and temperature. It is endemic to Algeria (Tindouf area) and Morocco (southwest region, more particularly the Souss area) [4,5]. In the southern parts of these two Maghreb countries, the argan tree is actually the last defense against desertification [6,7].

A previous study has characterized the structure of xylan and xylogucan-type hemicelluloses found in argan trees growing in Morocco [8]. Enzymatic treatment of these polysaccharides was conducted with an endo-(1-4)-β-d-xylanase (belonging to the glycoside hydrolase family 11) and with an endo-(1-4)-β-d-glucanase (belonging to the glycoside hydrolase family 7), respectively. Applied to xylan-type polysaccharides, the enzymatic hydrolysis generated oligomers whose structural units were determined as Xyl_(4-6)_-4-*O*-MeGlcA_(1-2)_. Applied to xyloglucan-type hemicelluloses, the enzymatic treatment revealed some typical XXXG-type xyloglucans but an original feature was also obtained with a β-d-Xyl*p*-(1→2)-α-d-Xyl*p* side chain linked to the cellotetraose sequence. Therefore, argan trees could potentially contain some original polysaccharidic structures. However, studies analyzing polysaccharides from Algerian argan trees are rare or non-existent. We have previously described the cell wall pectin isolated from the leaves of the argan tree, collected in Algeria [9,10]. To the best of our knowledge, the present study is the first report on cell wall-type xylan hemicellulose from the leaves of this species growing in this specific environment.

## 2. Results and Discussion

### 2.1. Extraction and Isolation of Structural Polysaccharides

The yield for each extract obtained from successive extractions of structural polysaccharides from argan leaves is listed in Table 1. The bulk material of the leaves consisted of molecules soluble in acetone/isopropanol, such as pigments. The resulting cell wall residue represented 51% of the dry mass of the leaves. The cellulosic extract (C6) comprised the main part of cell wall polymers by weight (0.86 g), accounting for 21.1% of the cell wall residue. The total weight of the hemicellulosic extracts was 0.75 g (18.5% of the cell wall residue) and that of the pectic extracts was 0.66 g (16.1% of the cell wall residue).

### 2.2. Monosaccharide Composition of the Hemicellulosic Extracts

The hemicellulosic fractions extracted with potassium hydroxide (S4) and sodium hydroxide (S5) were rich in xylose and arabinose (Table 2). The extracts also included lesser amounts of uronic acids, which accounted for 18.3% and 18.6% of the dry mass of the S4 and S5 extracts, respectively. The uronic acids contained small amounts of glucuronic acid (0.5%–0.6%), which indicated the extraction of (arabino)glucuronoxylan or the presence of 4-*O*-methyl-glucuronic acid residues, which are components of xylans. Nevertheless, the presence of xyloglucan polysaccharides could not be excluded, since the Xyl/Glu ratios found in the S4 and S5 extracts were respectively found equal to 4 and 5. These results are consistent with previous studies which reported the presence of these two kinds of hemicellulose in the leaves of Moroccan argan [8].

### 2.3. Infrared Analysis of the Hemicellulosic Extracts

Infrared spectroscopic analysis was conducted to compare the spectra of the two hemicellulosic extracts S4 and S5. The spectra exhibited absorption bands characteristic of xylans, between 1000 and 1100 cm^−1^, with a peak at about 1053 cm^−1^, corresponding to the stretching vibrations of the ring bonds and C–OH bonds [11]. An absorption band at about 1605 cm^−1^ indicated the presence of uronic acids in the form of acid salts, this band is partially covered up by an absorption band at 1630 cm^−1^, due to the presence of residual water. Additional bands detected at 1407–1415 cm^−1^ might indicate the presence of residual lignin, whose characteristic absorption peaks are at about 1410 cm^−1^ [12].

### 2.4. Purification and Characterization of KOH and NaOH Hemicellulosic Fractions

#### 2.4.1. Purification and Monosaccharide Composition

**Figure 1:**
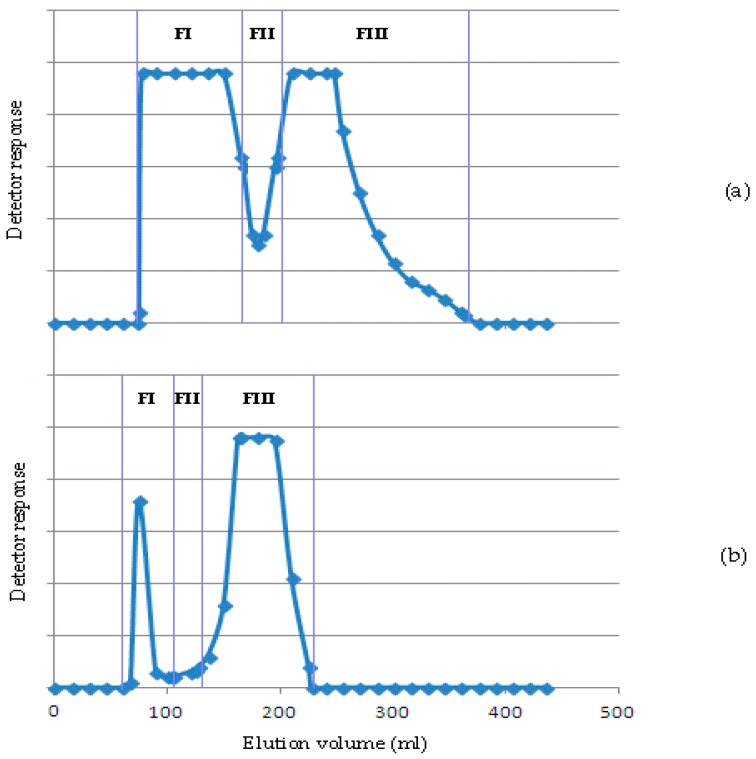
Sepharose CL-4B chromatography of (**a**) KOH-extracted hemicellulosic extract (S4); and (**b**) NaOH-extracted hemicellulosic extract (S5).

Typical chromatograms for the hemicellulosic polymers extracted with KOH and NaOH are shown in Figure 1.

We recovered the main fractions FI (the first elution fraction) and FIII (the third elution fraction) from the KOH-soluble hemicellulosic extracts (chromatogram in Figure 1a), and FI and FIII for the NaOH-extracted hemicellulosic extracts (chromatogram in Figure 1b). These fractions had the following dry weights: 142.8 mg, 63.0 mg, 37.0 mg, and 30.0 mg, respectively. All fractions contained significant proportions of uronic acids, between 17.1% and 30.8%. Analysis of the monosaccharidic composition of the main fractions demonstrated that xylose and arabinose were the predominant monosaccharides as they were present in all fractions collected (18%–30% xylose and 35%–43% arabinose). The presence of relatively small amounts of other monosaccharides such as rhamnose (less than 7%), glucose (8%), and trace amounts of glucuronic acid were also noted. Thus, the obtained fractions retained the characteristics of the starting extracts (Table 3).

#### 2.4.2. Oligosaccharide Fractions Obtained by Enzymatic Hydrolysis

The monosaccharidic composition of the oligosaccharide fractions derived from enzymatic hydrolysis of the hemicellulosic fractions is shown in Table 4. Xylose was the major monosaccharide present in all oligosaccharide fractions and accounted for 24%–58% of the total. Arabinose was present in large amounts and represented 20.4% of the KOH oligosaccharide fraction (FIII). The decrease in Ara content compared to the polysaccharidic fractions may be due to the substrate specificity of the enzyme, described as an endo-β-1-4-xylanase, which could be unable to hydrolyze xylan-type chains highly substituted with arabinose units [13]. Xyl/4-*O*-MeGlcA molar ratios equal to 10:1 have been found. These results are in agreement with those typically reported in the literature [14]. Such values are often encountered among dicotyledons, even if the MeGlcA content is extremely variable [15]. The Xyl/4-*O*-MeGlcA molar ratio can be larger than 20:1, such as in birch tree [16], where the insoluble hemicellulosic fractions isolated from the pericarp of the *Opuntia ficus-indica* fruit contain 12–65 xylose residues per uronic acid unit [17].

#### 2.4.3. Mass Spectrometry Analysis

The molecular weights and the degree of polymerization (DP) of the oligosaccharides present in the enzymatic hydrolyzates were determined by matrix-assisted laser desorption ionization-time of flight mass spectrometry (MALDI-TOF MS). The samples were studied in the native form. The results are shown in Table 5.

**Figure 2:**
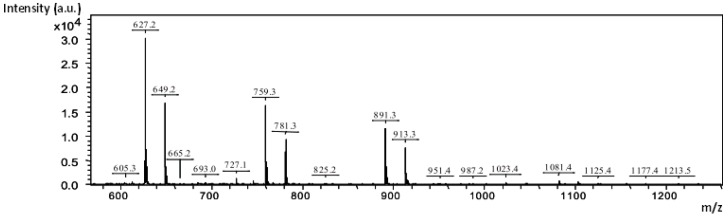
Mass spectrum from positive-mode MALDI-TOF MS for the FI KOH hemicellulosic fraction (1 mg·mL^−1^) generated by hydrolysis with endo-xylanase.

The chemical species in the different hydrolyzates identified with MALDI and Electrospray ionization (ESI) sources of ionization mainly corresponded to a series of pentose residues bearing a methylated uronic acid. Two species bearing two methylated uronic acids were identified by MALDI in positive mode (*m*/*z* 1081.4 and *m*/*z* 1213.5) (Figure 2).

The hydrolyzates of the different hemicellulosic fractions probably comprised arabinoxylo-oligosaccharide substituted with a methylated uronic acid unit, probably 4-*O*-methyl-glucuronic acid. They have been obtained by using an endo-β-(1→4)-xylanase originated from *Aspergillus oryzae*. This type of xylanase belongs to the GH-10 family which is known to require a low number of consecutive unsubstituted β-1,4-xylopyranosyl units in order to act on xylan chains [18], and is also known for its strong affinity for the links located close to the branch points of the main xylan chain [19]. It appears that the *Aspergillus oryzae* enzyme recognizes the glucuronosyl substituents and hydrolyzes the β-1,4-xylosyl linkage of the adjacent unsubstituted xylosyl residue in glucuronoxylans [20]. This endoxylanase does not seem to be strongly specific of a particular type of xylan, because it can hydrolyze hardwood glucuronoxylans, cereal arabinoxylans, as well as algal β-1,4-β-1,3-xylan [21]. The present results are consistent with this mode of action, which may explain the specific nature of the enzymatic hydrolysis that generates only oligosaccharides (one methylated uronic acid unit per six xylose/arabinose units). In addition, the current results showed some differences with those previously reported about xylan isolated from the leaves of Moroccan argan [8]: in the latter case, the enzymatic hydrolysate contained only traces of Ara and a high level of Xyl. Only the non-hydrolyzed-fraction of the polymer contained a high level of Ara (21%). That is why a xylan-type structure has been proposed for the hemicellulose of Morrocan leaf argan. A similar pattern has been described for alkali-soluble-xylans from the pericarp of prickly pear fruit [17] and from two species endemic to Central Africa [22].

The literature indicates that most 4-*O*-methyl-glucuronoxylans are isolated from tissues that contain secondary walls or mucilage. However, Dinand and Vignon (2001) [23] demonstrated, on the surface of cellulose microfibrils of sugar beet pulp, the presence of a 4-*O*-methyl-glucuronoxylan structure with similar rates of branching. These polysaccharides are considered to be constituents of the side walls and are generally infrequent in the primary wall.

Finally, an original structure has been obtained from the leaves of the Algerian argan tree. Alkali extractions followed by an endo-β-1-4-xylanase treatment have led to arabino-4-*O*-Me-glucuronoxylan-type oligosaccharides as confirmed by the monosaccharidic composition of oligosaccharidic fractions determined by GLC.

## 3. Materials and Methods

### 3.1. Biological Material

Argan tree leaves used in this study were collected in June 2010 from the Tindouf Province in southwestern Algeria. After collection, the leaves were dried in a ventilated oven (40 °C), ground (particle size < 200 µm), and stored in desiccators at room temperature.

### 3.2. Chemicals and Enzyme

All chemicals used were of analytical grade and were purchased from the following providers: Sigma (St. Louis, MO, USA), Acros (Morris Plains, NJ, USA), Alltech (Deerfield, IL, USA), Fluka (Buchs, Switzerland), Supelco (Bellefonte, PA, USA), Prolabo, and VWR (Fontenaysous Bois, France). Endo-β-(1→4)-xylanase isolated from *Aspergillus oryzae* (Novazymes, EC 3.2.1.8, Shearzyme 2X, GH-10) that were used to hydrolyze xylans were obtained from Sigma.

### 3.3. Isolation of Hemicellulose Fractions

**Figure 3:**
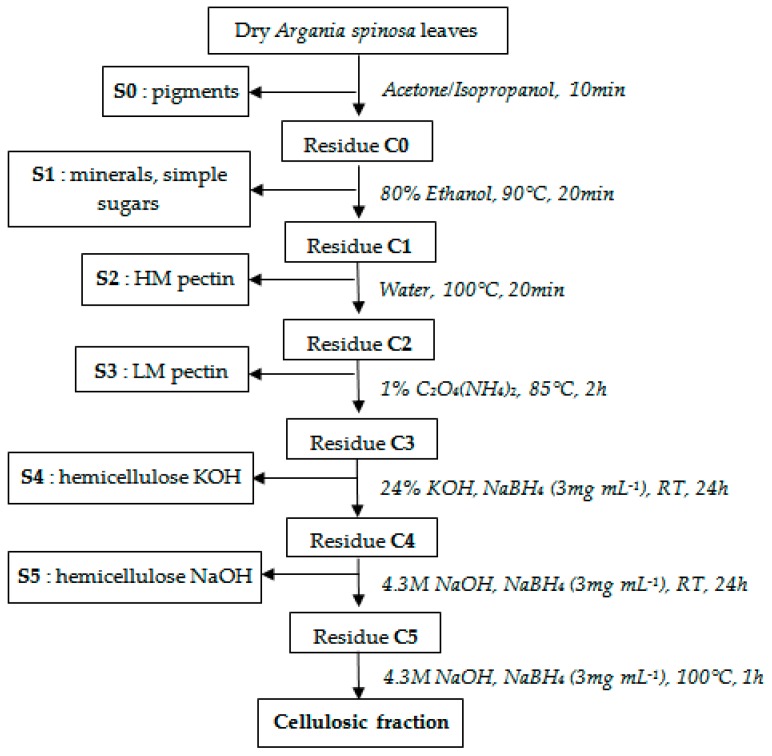
Extraction and isolation of polysaccharide fractions.

Dry *Argania spinosa* leaves (10 g) were treated sequentially with acetone/isopropanol (*v*/*v*) and 80% EtOH (20 min at 90 °C) to remove hydrocarbons, lipids, flavonoids, and oligomers. The alcohol insoluble residue (5.1 g) was depectinated by treatment with hot water (20 min at 100 °C), and 1% ammonium oxalate solution (2 h at 85 °C). The residue was then twice extracted, sequentially with 500 mL of 24% KOH and 4.3 M NaOH solutions containing NaBH_4_ (3 mg·mL^−1^) for 24 h. The different alkali extracts were neutralized by addition of glacial acetic acid and dialyzed against water (MWCO 6–8000 Da, Spectrapor, Rancho Dominguez, CA, USA). Hemicelluloses were finally precipitated by addition of three volumes of ethanol to the aqueous solution; the pellet was recovered after centrifugation, solubilized in water, and then freeze-dried and stored (Figure 3).

### 3.4. Purification of Hemicellulose Fractions

Hemicellulose fractions were purified by size-exclusion chromatography on Sepharose CL-4B (BIORAD-MWCO 30–5000 kDa; column Ø: 2.5 cm × 50 cm), and eluted at 15 mL/h with 50 mM sodium acetate solution, pH 5, at room temperature. The column effluent was monitored using a refractive index detector. The salts were removed from the recovered fractions by gel-permeation chromatography on a Bio-Gel P-2 column (Biorad; MWCO 100–1800 Da; column Ø: 2.5 cm × 70 cm) using water as the eluent to yield the purified hemicellulose soluble fractions.

### 3.5. Enzymatic Hydrolysis

Enzymatic hydrolysis was performed with an endo-β-(1→4)-xylanase (EC 3.2.1.8, Shearzyme 2X) at 55 °C for 1 h. The reaction was inactivated by the addition of three volumes of ethanol. The upernatant and precipitate were separated by filtration. The oligosaccharide hydrolyzate was then purified by size exclusion chromatography on Biogel P2. The elution profile was monitored by TLC using butanol/acetic acid/water (2/1/1, *v*/*v*/*v*) as the eluent. The revelation of spots was carried out by spraying orcinol-sulfuric acid reagent (0.001% of orcinol *m*/*v* in a 10% H_2_SO_4_ solution *v*/*v*) and heating at 100 °C.

### 3.6. Chemical Characterizations

#### 3.6.1. Chemical Composition of Extracts

Total sugar content of glucuronoxylans was measured by the phenol–sulfuric acid colorimetric method [24], using xylose as the standard; absorbance was read at 490 nm. Uronic acid content was determined colorimetrically at 520 nm by the *m*-hydroxydiphenyl method [25], with glucuronic acid as the standard.

#### 3.6.2. Monosaccharide Composition

Monosaccharide composition of polysaccharides was determined by gas–liquid chromatography, using a Perichrom gas chromatograph fitted with a flame-ionization detector. Monosaccharides were liberated from polysaccharides (200 µg) after methanolysis (MeOH/HCl 1 M, 24 h, 80 °C). Then the solutions (containing myo-inositol as the internal standard) were evaporated under nitrogen and 1 mL of methanol was added. Samples were defatted three times with 1 mL of heptane. After evaporation of the solutions, trimethylsilylation was carried out with BSTFA (*N*,*O*-bis-trimethylsilyl-trifluoroacetamide)-pyridine (1:1, *v*/*v*; 200 µL) at 27 °C for 2 h. Separation of the pertrimethyl-silylated methylglycosides was done using a CPSIL-5CB capillary column (Chrompack, 0.32 mm × 50 m), with the following temperature program; 120–240 °C at 2 °C·min^−1^. Nitrogen was the carrier gas at 0.5 atm.

#### 3.6.3. FT-IR Spectroscopy

Samples were characterized by Infrared (IR) spectroscopy with a 1000 FT-IR Perkin-Elmer Spectrum spectrometer in the 400–4000 cm^−1^ frequency range.

#### 3.6.4. Mass Spectrometry

Mass spectrometry analyses were conducted by the platform “Biopolymers-Interactions-Structural Biology” (INRA Research Unit 1268) (http://www.angers-nantes.inra.fr/plateformes_et_plateaux_techniques/plate_forme_bibs). Electrospray ionization ESI-MS and/or ESI-MS/MS data acquisition were performed on a Q-TOF Global mass spectrometer (Waters, UK). Samples were dissolved in H_2_O/MeOH (50:50) and introduced at a flow rate of 2 µL·min^−1^ in negative or in positive ionization mode. Matrix-assisted laser desorption/ionization (MALDI)-MS data acquisition was performed on a MALDI Autoflex III Smartbeam (Bruker Daltonics, Bremen, Germany). Samples were dissolved in H_2_O/Acetonitrile/DimethylAniline (50/50/0.2) at a concentration of 100 mg·mL^−1^.

## 4. Conclusions

Monosaccharidic analysis by GC and MALDI-TOF spectrometry was conducted to elucidate the composition and structure of xylans in the leaves of Algerian argan trees. The results confirmed that the main structure comprised arabino-4-*O*-methylglucurono-xylans and comparison with the litterature led to the proposal that the β(1→4) xylopyranose chain was partially substituted with α-l-Ara*f*-(1→3) residues and 2/3-substituted with uronic acid residues optionally methylated (4-*O*-methyl-glucuronic acid) with a degree of polymerization (DP) comprised between 4 and 7. To precisely identify their structure, the presently described oligosaccharidic fractions are being currently subjected to purification in view of further analysis by ^1^H- and ^13^C-NMR spectroscopy.

## Figures and Tables

**Table 1:** Yield (expressed in mass %) and composition of the cell wall extracts of argan leaves.

Fraction	Yield (Mass %)	UA (%)	NS (%)
**S0**: pigments	45.6 *	-	-
**S1**: minerals, simple sugars	3.5 *	25.1	74.9
**S2**: HM pectin	2.9 **	48.6	51.4
**S3**: LM pectin	13.2 **	67.9	32.1
**S4**: Hemicellulose KOH	11.8 **	18.3	81.7
**S5**: Hemicellulose NaOH	6.7 **	18.6	81.4
**C6**: Cellulose	21.1 **	-	-

* Weight % from the starting material; ** Weight % from the cell wall residue; UA: Uronic Acids; NS: Neutral Sugars.

**Table 2:** Monosaccharide composition determined by gas–liquid chromatography (GLC) of hemicellulosic extracts isolated from *A. spinosa*.

Glycosyl Residues	S4: Hemicelluloses KOH	S5: Hemicelluloses NaOH
Rha	7.2	5.8
Fuc	1.6	2.3
Ara	39.4	31.9
Xyl	24.9	33.3
Man	1.3	1.0
Gal	8.2	8.0
Glc	6.3	6.8
Gal A	4.9	6.4
Glc A	0.6	0.5
4-*O*-Me Glc A	5.6	4.0

**Table 3:** Monosaccharidic composition determined by GLC of hemicellulosic fractions obtained after Sepharose gel CL-4B chromatography.

Glycosyl Residues (Molar %)	S4: Hemicelluloses KOH		S5: Hemicelluloses NaOH	
	FI	FIII	FI	FIII
UA (%)	17.1	20.6	30.8	17.1
NS (%)	82.9	79.4	69.2	82.9
Rha	6.6	8.3	6.7	7.1
Fuc	1.2	2.2	2.5	4.3
Ara	35.8	43.0	35.4	36.1
Xyl	29.8	18.2	30.8	22.2
Man	0.9	0.8	0.7	1.7
Gal	6.3	10.8	6.2	8.6
Glc	8.4	6.0	7.4	8.3
Gal A	7.6	7.4	7.1	8.2
Glc A	0.5	1.2	0.5	0.6
4-*O*-Me Glc A	2.9	2.1	2.8	2.7

**Table 4:** Monosaccharide composition determined by GLC of oligosaccharides fractions derived from the hemicellulosic fractions by hydrolysis with endo-xylanase.

Glycosyl Residues	Oligosaccharides KOH Obtained by Enzymatic Hydrolysis		Oligosaccharides NaOH Obtained by Enzymatic Hydrolysis	
	FI	FIII	FI	FIII
Rha	4.3	3.3	4.3	5.2
Fuc	0.0	0.0	0.0	0.0
Ara	17.1	20.4	16.1	15.8
Xyl	58.1	45.8	24.0	31.6
Man	2.2	8.1	24.1	19.3
Gal	1.5	14.9	10.2	10.0
Glc	2.1	2.3	6.8	10.9
Gal A	5.4	0.3	9.1	3.9
Glc A	0.6	1.9	2.6	1.2
4-*O*-Me Glc A	8.7	3.0	2.8	2.1

**Table 5:** Positive-ion mode matrix-assisted laser desorption ionization-time of flight mass spectrometry (MALDI-TOF-MS) of the oligosaccharides generated from the hemicellulosic fractions by hydrolysis with endo-xylanase.

Subfraction Obtained by Enzymatic Hydrolysis	Compound	[M + Na]^+^
FI KOH	3(pentose) + 1(uronic acid methylated)	627.2
	4(pentose) + 1(uronic acid methylated)	759.3
	5(pentose) + 1(uronic acid methylated)	891.3
	6(pentose) + 1(uronic acid methylated)	1023.4
	5(pentose) + 2(uronic acid methylated)	1081.4
FIII KOH	3(pentose) + 1(uronic acid methylated)	627.2
	4(pentose) + 1(uronic acid methylated)	759.3
	5(pentose) + 1(uronic acid methylated)	891.3
FI NaOH	3(pentose) + 1(uronic acid methylated)	627.2
	4(pentose) + 1(uronic acid methylated)	759.3
FIII NaOH	3(pentose) + 1(uronic acid methylated)	627.2
	4(pentose) + 1(uronic acid methylated)	759.3
